# MiR-34 at the crossroads of SMA pathogenesis and therapy: Emerging biomarker and therapeutic target

**DOI:** 10.1016/j.omtn.2025.102557

**Published:** 2025-05-23

**Authors:** Jun-An Chen

**Affiliations:** 1 Institute of Molecular Biology, Academia Sinica, Academia Road Sec 2 No 128, Taipei 11529, Taiwan

## Main text

Spinal muscular atrophy (SMA) remains a devastating genetic disorder and the leading cause of infant mortality. It is linked to functional loss of the *survival motor neuron 1* (*SMN1*) gene. Restoration of SMN1 protein levels through antisense oligonucleotide (ASO) therapies or gene replacement has dramatically altered the therapeutic landscape.^1^ Nevertheless, significant challenges persist, namely: (1) the invasive nature of intrathecal delivery; (2) the prohibitive costs associated with treatment; and (3) the unclear biological basis underpinning variable patient responses, which likely reflect underlying disease heterogeneity. These concerns highlight an urgent need for authentic biomarkers capable of monitoring treatment efficacy and guiding precision medicine in SMA patients.^2^

Previous studies have proposed microRNAs (miRNAs) as potential biomarkers for motor neuron degeneration and related disease progression.^3^^,^^4^^,^^5^^,^^6^ However, these investigations were largely confined to serum-based analyses, in which systemic metabolic fluctuations unrelated to neuromuscular degeneration can obscure physiological interpretation. Moreover, prior efforts primarily identified associative, rather than causative, relationships between miRNA dysregulation and disease, leaving *bona fide* miRNA contributors to SMA pathogenesis elusive.

To address these limitations, two recent studies published in *Molecular Therapy - Nucleic Acids* employed multidisciplinary and systematic approaches to reveal a pivotal role for the miR-34 family in SMA pathology.^4^^,^^7^ In the study by Chen et al., a combination of in cellulo (mouse embryonic stem cells and human SMA patient-derived induced pluripotent stem cell spinal neurons) and *in vivo* (SMNΔ7 mouse model) platforms was employed to systematically identify spinal miRNA candidates. Among these, miR-34a emerged as the most consistently downregulated miRNA during early stages of motor neuron degeneration. Importantly, generation of *Mir34* family triple-knockout (TKO) mice (*Mir34a−/−*, *Mir34bc−/−*, *Mir449abc−/−*)^8^ recapitulated key SMA phenotypes, including postnatal lethality, axial hypotonia, reduced muscle fiber size, axonal swelling, and impaired neuromuscular junction architecture. Transcriptomic profiling of TKO spinal cords revealed that miR-34a preferentially targets genes involved in synaptic function, echoing two major pathological themes in SMA, i.e., sensory-motor circuit disruption and neuromuscular junction dysfunction. Furthermore, clinical analyses demonstrated that levels of miR-34 family peptides in cerebrospinal fluid (CSF) declined during the loading phase of nusinersen treatment in type I SMA patients, with lower baseline miR-34a/b levels correlating strongly with superior motor function outcomes one year later. These findings position miR-34a both as a potential therapeutic target and as part of a biomarker panel, together with phosphorylated neurofilament heavy chain, to monitor the treatment response.^4^

In parallel, Wu et al. arrived at a convergent yet nuanced conclusion. Utilizing a Taiwanese severe SMA mouse model with a lifespan of 10–11 days, they performed whole-transcriptome sequencing of the spinal cord, heart, and liver tissues at early postnatal stages.^7^ They uncovered widespread RNA dysregulation, including of 2,771 mRNAs, 1,633 long non-coding RNAs, 1,519 circular RNAs, and 382 miRNAs. Intriguingly, miR-34a was consistently upregulated across all tissues, most notably in the heart. Construction of competing endogenous RNA networks identified several novel miR-34a targets, including *Spag5*, lncRNA00138536, and circRNA007386, all implicated in cell cycle regulation. Functional assays confirmed that SMN depletion upregulated miR-34a, and its overexpression impaired cell cycle progression in both mouse myoblasts and human cardiomyocytes, mirroring the pathological transcriptome of SMA cardiac tissues. These findings broaden the impact of miR-34a beyond the central nervous system, underscoring its potential role in systemic manifestations of SMA.

Although both studies converge on miR-34a as a key regulator in SMA, they also reveal notable divergences. Chen et al. report miR-34a downregulation specifically in the spinal cord and CSF, emphasizing motor neuron-specific effects. In contrast, Wu et al. describe robust miR-34a upregulation, particularly in the heart and spleen, and to a lesser extent in the spinal cord. Such tissue-specific variability suggests that therapeutic strategies targeting miR-34a must be carefully tailored to avoid unintended consequences arising from systemic modulation. Furthermore, though AAV9-based delivery platforms show promise for miRNA therapeutics, their efficiency and specificity, particularly in non-neuronal tissues, require significant optimization before clinical translation.

Both studies collectively affirm the promise of miR-34a as a candidate biomarker for SMA therapy. Its consistent dysregulation and accessibility in CSF position it well for use in longitudinal disease monitoring. Baseline and dynamic changes in miR-34a levels during ASO treatment such as nusinersen offer predictive insights into patient responsiveness, potentially paving the way for personalized therapeutic strategies. Nevertheless, several critical questions remain. In particular, it will be important to (1) validate miR-34a findings in larger patient cohorts across SMA subtypes; (2) develop delivery methods capable of neuron- and/or tissue-specific modulation of miR-34a; (3) elucidate the full spectrum of miR-34a targets in neural versus non-neural tissues; and (4) investigate the long-term effects and safety profile of therapeutic miR-34a manipulation.

In conclusion, these two complementary studies elegantly establish miR-34a as a mechanistically relevant, clinically promising biomarker and therapeutic target in SMA. Future efforts should prioritize precision targeting approaches and multi-modal biomarker strategies to fully realize the translational potential of miRNA-based interventions in SMA.

## Declaration of interests

Jun-An Chen declared a provisional USA patent application for “Treating Spinal Muscular Atrophy (SMA) by modulating MiR34 and Use of MiR34 as a Predictive Biomarker of SMA” (*application no. 63/441,950*).

## References

[1] Finkel R.S., Mercuri E., Darras B.T., Connolly A.M., Kuntz N.L., Kirschner J., Chiriboga C.A., Saito K., Servais L., Tizzano E. (2017). Nusinersen versus Sham Control in Infantile-Onset Spinal Muscular Atrophy. N. Engl. J. Med..

[2] Wu Y.F., Chen J.A., Jong Y.J. (2025). Treating neuromuscular diseases: unveiling gene therapy breakthroughs and pioneering future applications. J. Biomed. Sci..

[3] Tung Y.T., Peng K.C., Chen Y.C., Yen Y.P., Chang M., Thams S., Chen J.A. (2019). Mir-17 approximately 92 Confers Motor Neuron Subtype Differential Resistance to ALS-Associated Degeneration. Cell Stem Cell.

[4] Chen T.H., Chang S.H., Wu Y.F., Yen Y.P., Hsu F.Y., Chen Y.C., Ming Y., Hsu H.C., Su Y.C., Wong S.T. (2023). MiR34 contributes to spinal muscular atrophy and AAV9-mediated delivery of MiR34a ameliorates the motor deficits in SMA mice. Mol. Ther. Nucleic Acids.

[5] Kaifer K.A., Villalón E., O'Brien B.S., Sison S.L., Smith C.E., Simon M.E., Marquez J., O'Day S., Hopkins A.E., Neff R. (2019). AAV9-mediated delivery of miR-23a reduces disease severity in Smn2B/-SMA model mice. Hum. Mol. Genet..

[6] Reichenstein I., Eitan C., Diaz-Garcia S., Haim G., Magen I., Siany A., Hoye M.L., Rivkin N., Olender T., Toth B. (2019). Human genetics and neuropathology suggest a link between miR-218 and amyotrophic lateral sclerosis pathophysiology. Sci. Transl. Med..

[7] Wu L., Sun J., Wang L., Chen Z., Guan Z., Du L., Qu R., Liu C., Shao Y., Hua Y. (2025). Whole-transcriptome sequencing in neural and non-neural tissues of a mouse model identifies miR-34a as a key regulator in SMA pathogenesis. Mol. Ther. Nucleic Acids.

[8] Chang S.H., Su Y.C., Chang M., Chen J.A. (2021). MicroRNAs mediate precise control of spinal interneuron populations to exert delicate sensory-to-motor outputs. eLife.

